# Admixture Mapping and Subsequent Fine-Mapping Suggests a Biologically Relevant and Novel Association on Chromosome 11 for Type 2 Diabetes in African Americans

**DOI:** 10.1371/journal.pone.0086931

**Published:** 2014-03-03

**Authors:** Janina M. Jeff, Loren L. Armstrong, Marylyn D. Ritchie, Joshua C. Denny, Abel N. Kho, Melissa A. Basford, Wendy A. Wolf, Jennifer A. Pacheco, Rongling Li, Rex L. Chisholm, Dan M. Roden, M. Geoffrey Hayes, Dana C. Crawford

**Affiliations:** 1 Charles Bronfman Institute of Personalized Medicine, Icahn School of Medicine at Mount Sinai, Vanderbilt University, Nashville, Tennessee, United States of America; 2 Center for Human Genetics Research, Vanderbilt University, Nashville, Tennessee, United States of America; 3 Department of Medicine, Division of Clinical Pharmacology,Vanderbilt University, Nashville, Tennessee, United States of America; 4 Department of Biomedical Informatics, Vanderbilt University, Nashville, Tennessee, United States of America; 5 Office of Personalized Medicine, Vanderbilt University, Nashville, Tennessee, United States of America; 6 Department of Pharmacology, Vanderbilt University, Nashville, Tennessee, United States of America; 7 Division of General Internal Medicine, Feinberg School of Medicine, Northwestern University, Chicago, Illinois, United States of America; 8 Division of Endocrinology, Metabolism, and Molecular Medicine, Feinberg School of Medicine, Northwestern University, Chicago, Illinois, United States of America; 9 Center for Genetic Medicine, Feinberg School of Medicine, Northwestern University, Chicago, Illinois, United States of America; 10 Division of Genetics, Children's Hospital Boston, Boston, Massachusetts, United States of America; 11 Office of Population Genomics, National Human Genome Research Institute, Bethesda, Maryland, United States of America; 12 Center for System Genomics, Department of Biochemistry and Molecular Biology, Pennsylvania State University, University Park, Pennsylvania, United States of America; Wake Forest School of Medicine, United States of America

## Abstract

Type 2 diabetes (T2D) is a complex metabolic disease that disproportionately affects African Americans. Genome-wide association studies (GWAS) have identified several loci that contribute to T2D in European Americans, but few studies have been performed in admixed populations. We first performed a GWAS of 1,563 African Americans from the Vanderbilt Genome-Electronic Records Project and Northwestern University NUgene Project as part of the electronic Medical Records and Genomics (eMERGE) network. We successfully replicate an association in *TCF7L2*, previously identified by GWAS in this African American dataset. We were unable to identify novel associations at p<5.0×10^−8^ by GWAS. Using admixture mapping as an alternative method for discovery, we performed a genome-wide admixture scan that suggests multiple candidate genes associated with T2D. One finding, *TCIRG1*, is a T-cell immune regulator expressed in the pancreas and liver that has not been previously implicated for T2D. We performed subsequent fine-mapping to further assess the association between *TCIRG1* and T2D in >5,000 African Americans. We identified 13 independent associations between *TCIRG1*, *CHKA*, and *ALDH3B1* genes on chromosome 11 and T2D. Our results suggest a novel region on chromosome 11 identified by admixture mapping is associated with T2D in African Americans.

## Introduction

In the United States 25.8 million people have diabetes, which accounts for 8.3% of the total U.S. population [1]. The distribution of individuals with type 2 diabetes (T2D) is disproportionate across race/ethnicity. In the United States, the burden of diabetes is higher in minority populations compared to European descent populations [2]. After adjusting for traditional T2D risk factors such as age, sex, body mass index (BMI), education, and physical activity; Native Americans, African Americans, and Latinos still have increased risk of T2D compared to European Americans [3]. These latter findings suggest that differences in genetic ancestry could possibly explain a proportion of the observed disparity of T2D across populations [4].

Over 30 novel loci have been identified by genome-wide association studies (GWAS) for T2D or related phenotypes [5]. These loci are common (allele frequency >25%) and confer small genetic effects (average odds ratio (OR) 1.06–1.7). The largest single SNP effect reported by GWAS is rs7903146, located in *TCF7L2*, which as originally identified by linkage analysis [6]. Other loci such as *KCNJ11* and *IRS1* were also replicated by GWA studies [7]–[10]. Some of the novel findings identified by GWAS for T2D include *KCNQ1*, *CDKN2A/CDKN2B*, *IGF2BP2*, and *SLC3OA8* [7], [9], [11], [12].

While all these studies have identified numerous risk loci for T2D in mostly European-descent populations, the role that genetic ancestry has on T2D risk is poorly understood. Common approaches to identify genetic risk factors such as candidate gene studies, GWAS, and linkage studies have failed to explain the racial/ethnic disparity of T2D. Alternative methods such as admixture mapping, which comprehensively identifies differences in genetic ancestry associated with disease risk in admixed populations, are needed to identify genetic loci responsible for ancestry-specific risk for T2D. Admixture mapping has successfully identified disease loci in African Americans for several common diseases such as end stage renal disease, white blood cell count, and prostate cancer [13]–[17]. Since T2D disproportionately affects admixed populations such as African Americans, using admixture mapping to identify potential disease loci is a promising tool to identify additional and possibly population-specific variants relevant to T2D risk.

Assessing the use of electronic medical records (EMRs) systems coupled to DNA repositories as a resource for genome science is one of the primary objectives for the National Human Genome Research Institute's electronic MEdical Records and GEnomics (eMERGE) Network [18]. Studies from eMERGE have demonstrated that EMR-based studies can replicate previously reported genetic associations [19]–[27] as well as discover novel associations [21], [23], [25], [28]. In the present study, we first performed a GWAS to generalize and/or replicate previous GWAS associations as well as identify novel disease loci for T2D in 1,563 African Americans (736 cases and 827 controls) from Vanderbilt (VGER) and Northwestern Universities (NUgene). Using admixture mapping, we then performed a genome-wide admixture scan to access whether genetic ancestry was associated in the same study population. We then fine-mapped significant loci identified from genome-wide admixture scan in a larger sample of 4,246 African Americans T2D cases (1,602) and controls (n = 2,644) from VGER. We show that using admixture mapping for discovery is a powerful approach for identifying disease loci for common diseases such as T2D in African Americans.

## Methods

### Ethics Statement

All samples included in the present study were ascertained at Vanderbilt Medical Center or Northwestern University. At Vanderbilt, DNA is collected from discarded blood samples remaining after routine clinical testing and is linked to de-identified medical records. According to the Vanderbilt Institutional Review Board (IRB) and the Federal Office of Human Research Protections provisions, the Vanderbilt protocol is considered nonhuman subjects research (The Code of Federal Regulations, 45 CFR 46.102 (f)) [29]. Subjects from Northwestern University are fully consented. Participants consent to the use of their clinical data stored in the electronic medical record (EMR) as well as their coded DNA samples for genetic research conducted by third party investigators [18]. Upon enrollment participants complete a detailed demographic questionnaire that is coupled with their DNA sample and longitudinal clinical data [18]. The Northwestern University Institutional Review Board approved the Northwestern site.

### Discovery Study Population

African American subjects were collected from the Vanderbilt and Northwestern University biobanks. The Vanderbilt biobank, BioVU, is a collection of DNA samples from discarded blood samples collected for routine clinical care linked to de-identified electronic medical records [30]. The Northwestern biobank, NUgene, combines DNA samples from consented participants with enrollment questionnaire and longitudinal data from the EMR [18].

Patients with available DNA samples were selected from BioVU and/or NUgene. BioVU subjects were African American as indicated by observer reported ancestry, which is highly concordant with genetic ancestry [31]. African American ancestry was self-reported for NUgene subjects [18], which is also known to be highly concordant with genetic ancestry [32]. Expert clinicians experienced with T2D diagnosis carefully designed our algorithm. T2D cases were defined as having the following in their EMR: a T2D ICD-9 medical billing code, information about insulin medication, abnormal glucose or HbA1c levels (≥100 mg/dl and ≥6.0%, respectively), or more than two diagnoses of T2D by a clinician. All T2D cases with an ICD-9 code for T1D were removed from further analyses [27]. All control subjects had to have at least 2 clinical visits, at least one blood glucose measurement, normal blood glucose or HbA1c levels, no ICD-9 codes for T2D or any related condition, no history of being on insulin or any diabetes related medication, and no family history of T1D or T2D [27]. Using these criteria, an automated method selected cases and matched study population demographics and differences between cases and controls are described in Table 1. There were 736 cases and 827 controls in our final study population for discovery.

**Table 1:** Discovery Study Population Characteristics.

Discovery Study Population (N = 1,563)			
Variable	Cases (n = 736)	Controls (n = 827)	P-value*
Mean Age (yrs.)	53 (±13.41)	43 (±14.55)	<0.00001
% Female	62%	67%	<0.04
Mean % European Ancestry	20.3% (±11.64)	20.6% (±14.26)	0.36

Fine-Mapping Study Population (N = 4,246)			
Variable	Cases (n = 1,602)	Controls (n = 2,644)	P-value
Mean Age (yrs.)	52 (±15.83)	46 (±18.84)	<0.00001
% Female	67%	67%	0.23

Global ancestry was determined using 4,333 autosomal ancestry informative markers (AIMs) on the Illumina 1M BeadChip (Tandon et al., 2011).

*Student's T-test or χ^2^ performed in STATA.

### Fine-mapping Study Population

Since BioVU accrues new samples each week, we re-ran the algorithm used for the discovery analysis for this follow-up study in June of 2011 (∼a year later). We identified a total of 1,659 cases and 3,372 controls for this fine-mapping study, of which 1,272 samples overlap with the discovery study. Study population characteristics are described in Table 1. All subjects were observer-reported African American, at least 18 years of age, and were collected from the Vanderbilt University biobank, BioVU. The algorithms used to define cases and controls are consistent with those used in the discovery population.

### Genotyping and SNP selection

For the GWAS and genome-wide admixture scan, all individuals that met the inclusion criteria were genotyped for >1.1 million SNPs using the Illumina 1M BeadChip at the Broad Institute. Data were cleaned by the eMERGE QC pipeline [33]. We checked for individuals with cryptic relatedness, ancestry inconsistent with observer- or self-reported ancestry, anomalous X-chromosome heterozygosity or poor genotyping efficiency and removed such individuals from future analyses (n = 46). Markers used as intensity probes, had technical failures, were monomorphic, had genotyping efficiencies <99%, had discordant calls with duplicates, had a Hardy Weinberg Equilibrium (HWE) p-value<1×10^−4^, and had ≥1 Mendelian errors were removed. We tested SNPs that were out of HWE for an association with T2D; however, our significant results are identical regardless of this inclusion. A total of 930,000 SNPs remained for association analyses after quality control.

For fine-mapping we genotyped 51 tagSNPs approximately 100 kb upstream and downstream of the *TCIRG1* gene that we identified by admixture mapping. HapMap Yoruba population linkage disequilibrium (LD) patterns were used to select tagSNPs in this region (http://pga.gs.washington.edu/). All tagSNPs with a minor allele frequency >0.08 were selected for this analysis. For quality control purposes, we purposely included 20 (out of 1,276) SNP pairs that are in high LD (r^2^>0.80) with each other. Genotyping was performed at the Vanderbilt University Center for Human Genetics Research DNA Resources Core using Sequenom based on a single-base primer extension reaction coupled with mass spectrometry. Common genotyping quality control metrics such as allele frequency and HWE were assessed as described above resulting in a total of 48 SNPs tested in our fine-mapping analysis.

### Statistical Methods

We first performed a genome wide association study (GWAS) with >900,000 SNPs genotyped on the Illumina 1M Bead Chip. Assuming an additive genetic model, we performed single SNP tests of association in 736 T2D cases and 827 controls (n = 1,563) using logistic regression in PLINK [34]. All tests were adjusted for age and sex.

Using our GWAS genotype data we also performed an admixture scan on a subset of 4,333 autosomal ancestry informative markers (AIMs) on the Illumina 1M BeadChip [35]. In this study we used ANCESTRYMAP to report global estimates of European ancestry for each individual and to identify disease loci throughout the genome. We tested AIMs for disease risk variants that differ in frequency across ancestral populations in this African American study population [36]. We calculated ancestral allele frequency for all of the markers tested in two HapMap II reference populations, CEU and YRI. We also assumed a prior risk distribution of 1.2 (required for ANCESTRYMAP) with 100 burn in and 200 follow on iterations. Current admixture mapping software including ANCESTRYMAP does not allow for covariates in the model; therefore we were not able to adjust for age and sex, which might confound our analysis. ANCESTRYMAP uses two statistics to determine disease association: the genome-wide LOD score (LGS, >2.0 significant), the locus–specific LOD score (>5.0 significant, >4.0 suggestive), and the case-control statistic (CCS, >5.0, significant) [36].

Pairwise LD (r^2^) was calculated and plotted using Haploview [37]. Power calculations were performed using Quanto [38].

For fine-mapping, we performed single SNP tests of association assuming an additive genetic model for SNPs with the *TCIRG1* that passed QC in PLINK [34]. Analyses were performed unadjusted and adjusted for age and sex. All results were plotted using Synthesis-View [39]. To account for multiple testing, we employed Bonferroni correction and declared a significance threshold of p<0.001. LD was calculated by measuring the correlation coefficient r^2^ in Haploview [37].

## Results

### Discovery: GWAS

**Figure 1:**
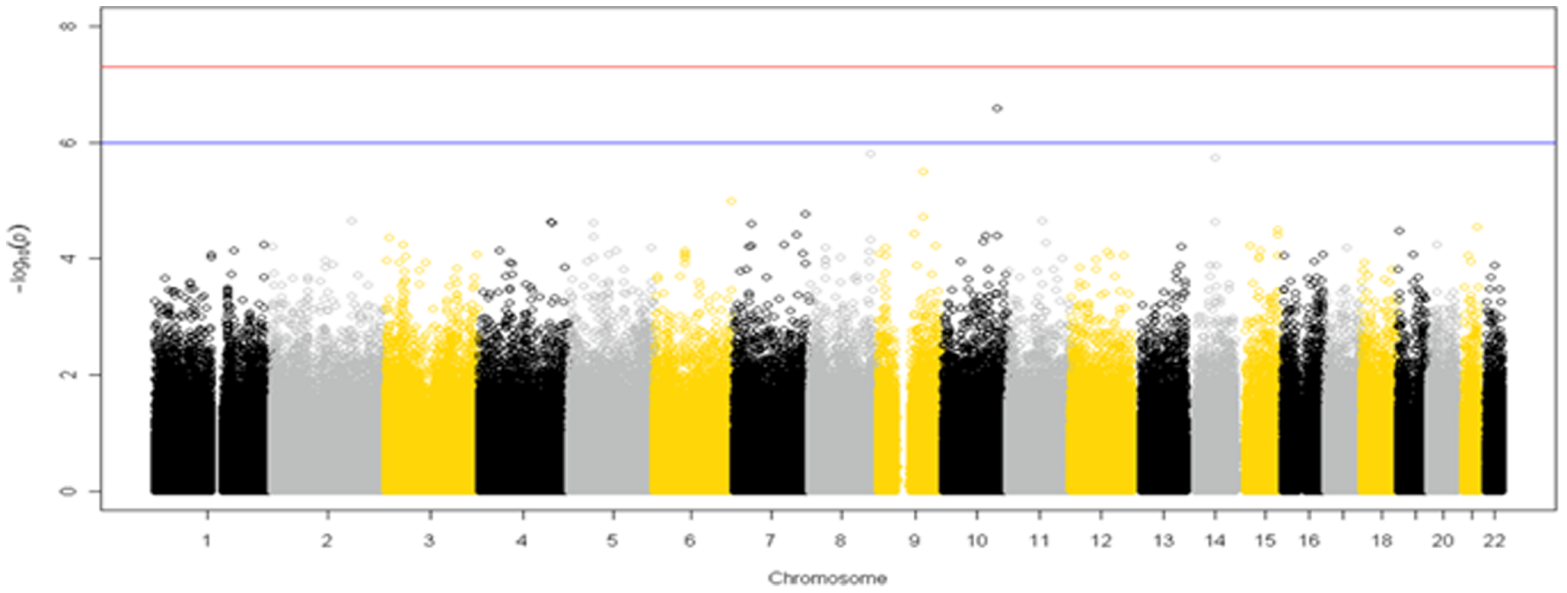
Genome-wide association results for T2D in African Americans from VGER/NUgene. We performed single SNP test of association with >930K SNPs across the genome using logistic regression in 736 T2D cases and 827 controls. The red line indicates the genome-wide significance level p<10^−8^ and the blue line is indicate of suggestive associations at p<10^−6^.

We first performed a GWAS of >900,000 SNPs in 1,563 African Americans from VGER/NUgene. As previously reported, only one SNP (rs7903146) met genome-wide significance at p<10^−8^ (OR = 1.7, p = 1.17×10^−8^) in this African American study population [27]. This intronic SNP is located on chromosome 10 in the transcription factor 7–like 2 (*TCF7L2)* gene and has been previously identified with an association with T2D and related phenotypes [7], [9]–[12], [27], [40]. Three additional SNPs were significant at the p<10^−6^ level (Figure 1). Intergenic SNP rs9347819, located on chromosome 6, was associated with increased risk of T2D (OR = 1.9, p = 5.63×10^−7^). Intronic *PRUNE2* rs11177982 located on chromosome 9 was also associated with increased risk for T2D (OR = 2.15, p = 7.112×10^−6^). One SNP (rs1048317) located in the intron of *LARGE* on chromosome 22, was less frequent in cases compared to controls. There were no other SNPs that reached genome-wide significance at p<10^−8^ or suggestive significant at p<10^−6^.

### Discovery: Genome-wide Admixture Mapping

**Figure 2:**
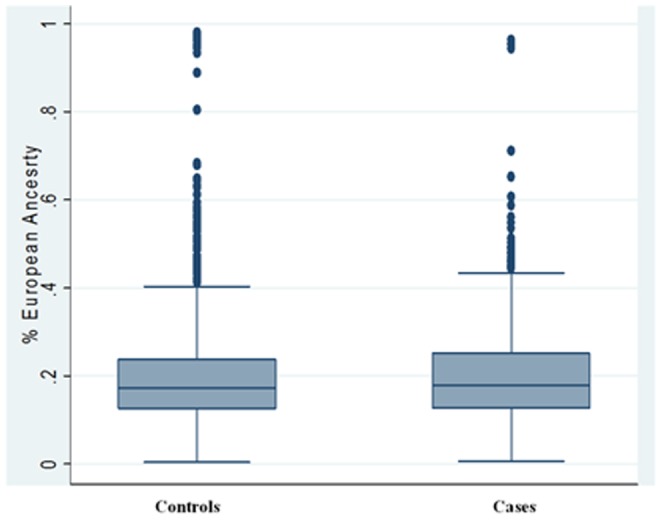
Comparison of global European ancestry in African American T2D cases and controls. Genome-wide estimates of percent European ancestry were calculated for each individual using ANCESTRYMAP. Average European ancestry was compared between cases (20.3%) and controls (20.6%, p = 0.72).

We performed a genome-wide admixture scan on a subset of 4,016 autosomal ancestry informative markers (AIMs) that passed QC and were genotyped on the Illumina 1M Bead Chip using ANCESTRYMAP [35]. Global estimates of European ancestry across the genome were calculated for T2D cases and controls. There were no differences in global European ancestry between cases and controls (p = 0.71). On average cases and controls have 20.3% and 20.5% of their genome from European ancestry, respectively (Figure 2).

**Figure 3:**
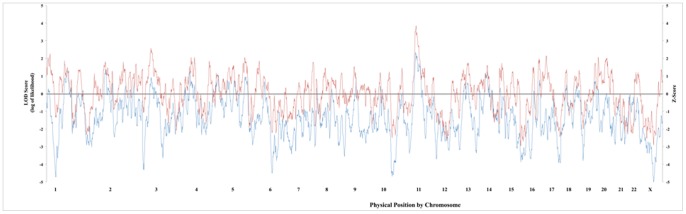
Genome-wide admixture Scan for Type 2 Diabetes. The log (base 10) of the likelihood, referred to as the locus genome statistic, is plotted on the y-axis (in blue) and represents the likelihood of being a disease locus vs. non-disease locus dependent on significant differences in ancestral allele frequencies. The case-control statistic for each SNP is plotted on the secondary y-axis (in red) and is represented as a Z-score.

In addition to global admixture estimates, we also sought to localize T2D risk variants in this African American study population. We tested AIMs across the entire genome for variants with a higher percentage of ancestry from one ancestral population in cases compared to controls. The global LGS across the all autosomes was 0.05 indicating most loci in the genome were not associated T2D status in this study population. The lowest LGS was −0.89, on chromosome 7, and the most significant region in the genome was on chromosome 11 and had a LGS of 2.30. Variants in this region had a local LGS ranging from 2.05 to 2.34 and a CCS ranging from 3.2 to 3.7 (Figure 3). One SNP, rs308328, had the highest test statistic (LGS = 2.34, CCS = 3.7), which is located in an intron of *UNC93B1* (Figure 3). This gene has no known function in humans; however, it is about 50 kb upstream of *TCIRG1*, which is mainly expressed in T-cells and nominally expressed in the pancreas [41] http://biogps.org/#goto=genereport&id=10312).

### Fine-mapping

**Figure 4:**
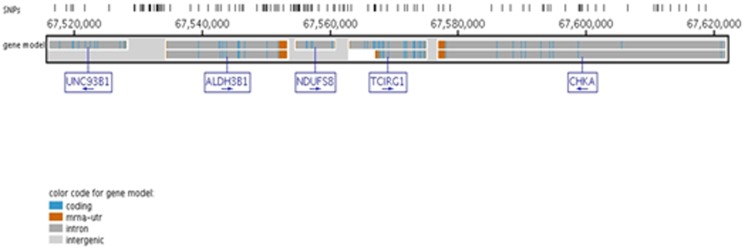
Candidate region targeted for fine-mapping. Using Seattle SNPs genome browser, the candidate genes located within 100*TCIRG1* gene, their orientation, and gene structure are displayed. SNPs annotated for these genes are located at the top of the figure denoted by hash marks. (Image generated from http://pga.gs.washington.edu/).

Loci identified by admixture mapping are usually large in size making it difficult to identify casual variants without fine-mapping. Our most significant signal is located on chromosome 11 and has strong biological implications since it is expressed in the pancreas. The locus is approximately 28,000 kb in size and includes over 90 genes making it unclear which gene is associated with T2D and warrants fine-mapping (Figure 3). We examined this signal on chromosome 11 in our discovery GWAS data to determine if the effect we observe in our admixture scan was significant in our GWAS. There were 29 SNPs in this region that were also genotyped in the discovery GWAS; however, none reached genome-wide significance or suggested an association at p<10^−6^. In an attempt to increase our power and extensively examine the region on chromosome 11 we performed fine-mapping for the *TCIRG1* region (100 kb upstream and downstream) with selected tagSNPs from Hap Map YRI in ∼5,000 T2D African American cases and controls from BioVU (Figure 4). We detected seven novel associations near the *TCIRG1* candidate region identified by admixture mapping that met our conservative Bonferroni significance threshold of p<0.001, all of which were located in the *CHKA* gene. Having one or more copies of the minor allele increases the risk of having T2D in African Americans for six of these SNPs (OR = 1.08–1.37). One SNP (rs10791957) was common in this African American dataset (MAF = 0.41) and was associated with decreasing the risk of T2D (OR = 0.83). There were an additional twelve SNPs within this region that were significant at the p<0.05 level, suggestive of an association. There were seven SNPs in the *CHKA* gene, three SNPs in the *ALDH3B1*, and two SNPs in the *TCIRG1* that trended towards significance (Table 2). Of the twelve associations, having one or more copies of the minor allele decreases the risk of T2D for half (six SNPs) while the other half increases the risk of T2D in African Americans (Table 2).

**Table 2:** Fine-mapping association results.

SNP	Gene/location	Coded Allele	OR	95% CI	SE	P
rs105147	*ALDH3B1* intron	A	0.89	(0.80, 1.00)	0.06	0.050
rs2286164	*ALDH3B1* intron	C	1.09	(0.98, 1.22)	0.05	0.100
rs2286169	*ALDH3B1* intron	G	1.15	(1.03, 1.29)	0.06	0.014
rs308335	*ALDH3B1* intron	G	0.95	(0.82, 1.09)	0.07	0.455
rs308337	*ALDH3B1* intron	A	0.94	(0.81, 1.09)	0.07	0.404
rs308338	*ALDH3B1* intron	G	0.93	(0.84, 1.04)	0.05	0.204
rs557098	*ALDH3B1* intron	G	0.96	(0.83, 1.10)	0.07	0.562
rs2286163	*ALDH3B1* Ser→ Ser	A	1.16	(1.03, 1.31)	0.06	0.013
rs11825872	*ALDH3B1*downstream	C	0.90	(0.73, 1.10)	0.10	0.302
rs3751082	*ALDH3B1*Leu→Leu	T	1.07	(0.93, 1.23)	0.07	0.315
rs12418774	*ALDH3B1*upstream	G	1.16	(0.89, 1.51)	0.14	0.280
rs3763942	*ALDH3B1*upstream	C	1.00	(0.83, 1.19)	0.09	0.976
rs7928739	*CHKA* intron	A	0.87	(0.78, 0.97)	0.06	0.011
rs3794186	*CHKA* 3′ UTR	T	1.05	(0.87, 1.26)	0.09	0.636
rs11228145	*CHKA* downstream	T	0.87	(0.76, 1.00)	0.07	0.052
rs1547888	*CHKA* downstream	C	1.17	(1.03, 1.32)	0.06	0.016
rs1547889	*CHKA* downstream	T	1.14	(0.99, 1.30)	0.07	0.062
**rs2511469**	***CHKA*** ** downstream**	**A**	**1.21**	**(1.09, 1.34)**	**0.05**	**4.46E-04**
rs2511470	*CHKA* downstream	C	1.14	(1.01, 1.27)	0.06	0.029
rs2511472	*CHKA* downstream	C	0.87	(0.78, 0.97)	0.06	0.010
rs2512623	*CHKA* downstream	T	0.86	(0.77, 0.95)	0.06	0.005
**rs7944372**	***CHKA*** ** downstream**	**C**	**1.23**	**(1.11, 1.37)**	**0.05**	**1.04E-04**
**rs10791957**	***CHKA*** ** intron**	**A**	**0.83**	**(0.74, 0.92)**	**0.05**	**4.71E-04**
**rs2511437**	***CHKA*** ** intron**	**G**	**1.20**	**(1.08, 1.33)**	**0.05**	**5.52E-04**
rs2511439	*CHKA* intron	T	1.12	(0.96, 1.31)	0.08	0.159
**rs2512612**	***CHKA*** ** intron**	**C**	**1.22**	**(1.10, 1.36)**	**0.05**	**1.61E-04**
**rs4930557**	***CHKA*** ** intron**	**C**	**1.22**	**(1.10, 1.35)**	**0.05**	**2.18E-04**
rs6591331	*CHKA* intron	A	1.15	(1.03, 1.28)	0.06	0.016
**rs6591333**	***CHKA*** ** intron**	**G**	**1.20**	**(1.08, 1.34)**	**0.05**	**8.88E-04**
rs7123035	*CHKA* intron	A	0.97	(0.82, 1.15)	0.08	0.739
rs7952122	intergenic (*UNC93B1 ALDH3B1*)	A	1.04	(0.93, 1.16)	0.06	0.497
rs308351	LOC100132261 downstream	C	1.05	(0.92, 1.20)	0.07	0.436
rs308309	LOC100132261 upstream	G	0.93	(0.79, 1.09)	0.08	0.362
rs4147776	*NDUFS8* 5′ UTR	C	1.18	(0.88, 1.58)	0.15	0.277
rs11824781	*NDUFS8* intron	C	0.97	(0.84, 1.12)	0.07	0.676
rs3115545	*NDUFS8* intron	T	0.94	(0.77, 1.14)	0.10	0.532
rs10896288	*NDUFS8* upstream	G	0.95	(0.82, 1.09)	0.07	0.448
rs11823975	*NDUFS8* upstream	C	0.88	(0.73, 1.05)	0.09	0.165
rs3115546	*NDUFS8*intron	G	0.94	(0.83, 1.06)	0.06	0.332
rs7949541	*NDUFS8*intron	T	1.01	(0.80, 1.28)	0.12	0.931
rs3133269	*NDUFS8*upstream	C	1.01	(0.90, 1.13)	0.06	0.821
rs7951010	*TCIRG1* upstream	T	0.93	(0.76, 1.14)	0.10	0.479
rs884826	*TCIRG1* upstream	A	0.87	(0.78, 0.97)	0.06	0.015
rs2075609	*TCIRG1* intron	G	1.16	(1.05, 1.29)	0.05	0.004
rs2240387	*UNC93B1*	G	1.05	(0.86, 1.13)	0.06	0.380
rs3808969	*UNC93B1*downstream	C	1.03	(0.87, 1.22)	0.09	0.757
rs308328	*UNC93B1*intron	A	0.98	(0.86, 1.13)	0.07	0.796

We performed logistic regression with 48 SNPs within 100 kb of *TCIRG1* gene with T2D case/control status in 4,246 African Americans. All tests were adjusted for age and sex, assuming an additive model. Bolded are Bonferroni corrected significant associations at p-value<1.0 E^−3^.

**Figure 5:**
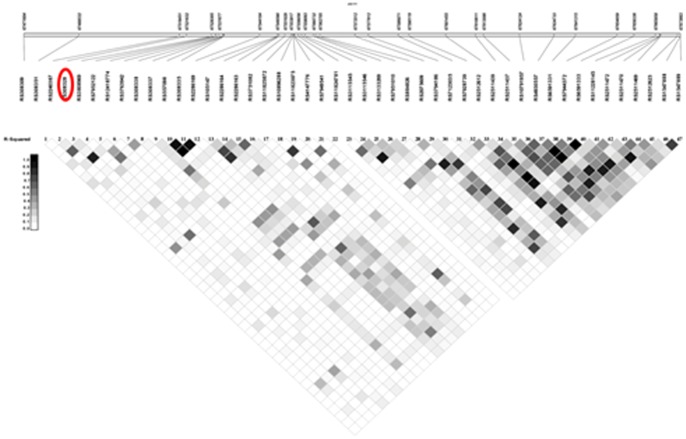
Linkage disequilibrium 100 kb flanking rs308328 in African American T2D cases and controls. We calculated pairwise LD (r^2^) around our most significant finding (circled in red) from the admixture scan in this African American study population using Haploview [37].

As previously mentioned, we selected several SNPs in high LD for quality control purposes. To distinguish between independent and redundant results, we calculated pairwise LD between all SNPs in this African American study population (Figure 5). There were a total of 22 highly correlated SNP-SNP pairs (r^2^>0.80), 20 of which were expected based on our assay design (Figure 5). Taking into account these correlations, there were only two independent loci within this area (r^2^<0.80) that met our significance threshold.

## Discussion

We successfully identified several associations with T2D in African Americans using samples derived from a biorepository linked to EMRs. Using conventional GWAS, we replicated a known association, rs7903146 (*TCF7L2*), in this African American study population consistent with several T2D studies in African Americans and with the work of others using this study population [42]. Similar to European populations (MAF = 0.29, OR = 1.26) [43], this SNP is common in African Americans (MAF = 0.30) and is associated with T2D risk (OR = 1.69).

In addition to confirming the *TCF7L2* locus with T2D in African Americans, using admixture mapping, we also identified a potential novel region on chromosome 11 that is biologically relevant with T2D risk. This region included five genes; one of which *TCIRG1* is nominally expressed in the pancreas although not yet associated with T2D from existing GWA studies or in our original GWAS in African Americans. However the locus on chromosome 11 is large and while we were able to narrow down the region to 50 kb around the most significant variant, the casual variants and/or genes were not explicitly identified. Subsequent fine-mapping of the *TCIRG1* locus confirmed a total of seven significant associations in this region at p<0.001, two were in *TCIRG1* at the p<0.05 level.

It is likely that we were underpowered to detect the *TCIRG1* association in our discovery GWAS analysis since we assumed there were no differences in local ancestry between cases and controls. Using a two-stage approach, admixture mapping coupled with fine-mapping, for discovery is potentially a more robust approach. This approach tests two hypotheses: (1) the presence of a disease locus due to differences in local ancestry compared to local estimates assuming disease allele frequency is equal in cases and controls (admixture mapping) and (2) the presence of a disease locus when there are known differences in local ancestry present and the disease allele frequency differs between cases and controls (fine-mapping the admixture mapping signal).

The admixture peak on chromosome 11 was large and included over 90 genes making it difficult to identify the casual locus. Our most significant finding from the admixture scan, rs308328, is located in the *UNC93B1* gene. This gene encodes the 12 membrane spanning protein UNC-93B and is involved in the exogenous antigen presentation and the signaling of toll-like receptors [44]. Mutations in *UNC93B1* in humans have led to impaired production of interferon which is necessary to fight herpetic virus infection [45]. To our knowledge this gene has not been associated nor has any functional relationship with T2D. *UNC93B1*, however, is located upstream of *TCIRG1* which is highly expressed in T cells and nominally expressed in the pancreas [41] (http://biogps.org/#goto=genereport&id=10312). While this region is gene rich, there are only 146 common SNPs reported in Yoruban (YRI) HapMap samples within 100 kb of the *UNC93B1* gene [46]. To determine if rs308328 is correlated with SNPs in the *TCIRG1* gene and thus representing this effect, we calculated pairwise LD (r^2^) in this region (Figure 5). There were only 59 SNPs in this region genotyped in our study population, and there were high levels of LD in our African American study population (Figure 5). However there is only one SNP (rs308351) located in an intergenic region, which is in moderate LD (r^2^<0.80 and >0.40) with rs308328 in our dataset.

Previous admixture mapping results in African Americans for obesity and T2D have been performed [47], [48]. We were unable to replicate association from these studies for several reasons. The admixture scan for obesity revealed two signals on chromosome X and one on chromosome 5 associated with BMI [47]. We failed to detect these signals in the present study, which is likely due to a combination of being underpowered due to sample size and differences in study design. The study conducted by Cheng et al. used a small subset of 1,400 ancestry informative markers (AIMs) to identify regions in the genome that vary in ancestral allele frequency. It is likely that the *TCIRG1* region in addition to many regions throughout the genome, were not covered using these markers. Thus we were not surprised that the *TCIRG1* was not identified in the Cheng et al. analysis. In addition to differences in SNP selection, we believe we were unable to replicate the finding by Cheng et al. due to differences in phenotype definition. In contrast to the Cheng et al. analysis that sought to identify genetic variants associated with dichotomized BMI, an intermediate phenotype, we tested for genetic variants associated with a well-defined end phenotype, T2D. However, BMI and T2D are highly correlated making it plausible that the associations we report could in fact be associated with BMI and/or T2D, a limitation to current admixture mapping methods.

The other admixture scan tested for an association with T2D in African Americans also identified two regions on chromosome 12 and one suggestive peak on chromosome 13 associated with T2D in African Americans [48]. As previously stated, we failed to detect these signals in this study. Unlike the admixture scan performed for BMI, this analysis used T2D case/control status and the failure to replicate their finding in our study was unexpected. However, there are still significant differences in phenotype definition and study design compared to the present work. Defining phenotypes from electronic medical records can be a cumbersome task for many reasons. First, all of the samples in the present study were extracted from a clinical population linked to electronic medical records. In comparison, the studies accessed by Cheng et al. [48] were three traditional epidemiologic studies (Atherosclerosis Risk in Communities, Jackson Heart Study, and Multi-ethnic Cohort Study), all of which defined T2D at baseline using a combination of labs (such as blood glucose), self-reported medication history, and self-reported T2D history. Given the dense clinical data available and the longitudinal nature of the electronic medical record system, it is likely that our definition of T2D in the present study is more stringent and therefore varies significantly compared to Cheng et al. [48]. Another possible reason we fail to replicate the findings of Cheng et al. may be due to s difference in sample size and power (Cheng et al. n∼7,000 and present study n = 1,500). To date the associations reported by Cheng et al. have yet to be replicated in an independent cohort [48].

We sought to fine-map the *UNC93B1* region identified by our admixture scan (Figure 4). We were able to identify several associations in this region; however, there were no significant findings in the *TCIRG1* or the *UNC92B1* genes that met our Bonferroni significance threshold (Table 2). In fact all of the significant associations were located in the choline kinase alpha (*CHKA*) gene, which encodes a protein essential in the phospholipid biosynthesis and may contribute to tumor cell growth [49], [50].

There were several limitations to the present study. For our GWAS we were underpowered to detect effects smaller than 1.6 (OR) for common SNPs (MAF >0.05). Our study population compared to recently published GWAS for T2D [51] is relatively small and underpowered. Furthermore, most GWAS fixed-content products are biased to common variation and based on LD patterns observed in European populations [52]. Rare variation and/or population-specific variation are currently underrepresented in GWAS fixed-content products. These limitations could have an impact on our ability to detect associations for T2D in diverse populations.

Metabolic diseases, such as T2D have a complex disease etiology and are likely caused by a combination of several environmental and genetic risk factors. Increased body mass index (BMI), as a result of poor diet and a sedentary lifestyle, is the major risk factor for T2D [53]. Under the clinical definition of obesity (BMI >30), 85% of T2D cases are obese [54]. We were not able to adjust for BMI in this work due to missing data and limitations of the current admixture mapping methods. In the present analysis >55% of the study population were missing BMI measurements and as previously mentioned current admixture mapping methods do not allow for covariates to be incorporated in the model. Given the correlation with BMI and T2D, we caution the interpretation of the results presented in this work, as the association we identified might also be associated with BMI.

The admixture scan was limited to ancestry informative markers (AIMs) genotyped on the Illumina 1M. A total of 4,016 AIMs were used, which only represents 0.37% of the markers genotyped in the entire dataset. The AIMs were previously identified for several GWAS platforms by Tandon et al. [35]. Furthermore, it is possible that there are several SNPs genotyped on these platforms that do not meet the criteria outlined by Tandon et al. but are informative and possibly associated with T2D status [35]. In the present analysis we did include SNPs that failed to meet the criteria outlined by Tandon et al. in our admixture scan.

The process by which SNPs were selected for the fine-mapping analysis limits our power to detect rare variants. We limited the fine-mapping study to one gene region from approximately 90 genes located within the admixture peak. We based our gene selection on known biological function and presumed relevance to T2D, but it is possible that other genes within the admixture peak are responsible for the association with T2D. We implemented a tagSNP approach to select specific SNPs for genotyping. The use of tagSNPs is a cost effective alternative to genotyping all known common variants regardless of linkage disequilibrium patterns throughout the genome. The caveat is that there is a possibility that the associated SNPs are not the functional or true “risk” SNP but are in linkage disequilibrium with the un-genotyped functional variant. Also, a fraction of SNPs must be assayed directly as no SNPs will serve as sufficient tags or proxies. We selected common tagSNPs with a minor allele frequency of at least 0.08 within 100 kb of the *TCIRG1* gene. Selected SNPs had to have an r^2^>0.80 with other SNPs in the region to be selected for this analysis. By limiting our analysis to only common tagSNPs we fail to capture rare variants, which may have larger effects on T2D risk. Additionally our high r^2^ threshold resulted in several redundant associations due to moderate to high LD in this gene region. Finally, it is possible that we failed to assay a sufficient proxy or tagSNP for the true “causal” variant associated with T2D.

This work emphasizes the importance of genetic association studies in African American populations. Compared to our discovery analysis, which was powered to detect moderate to large associations (≥1.7 OR), the fine-mapping study was sufficiently powered (>80%) to detect effects as small as 1.3 (OR) given our sample size. Furthermore, fine-mapping the *TCIRG1* improved our power to directly detect an association in this candidate region. Of the 51 SNPs selected for fine-mapping only 29 were directly assayed and genotyped in the original GWAS. We identified eight independent associations at p<0.05, five were also included in the GWAS. None of these SNPs were significant in our GWAS (p<0.05), which might be due to an increase in power (larger sample size) but also indicating that admixture mapping followed by fine-mapping is a powerful method in the identification of novel disease loci. Overall, these data contribute to the understanding of the genetic etiology of T2D in African Americans and serve as a starting point for future sequencing and molecular studies to better understand the biological processes that lead to T2D and other complex human diseases.
